# Glyoxalase 1 Inducer, *trans*-Resveratrol and Hesperetin–Dietary Supplement with Multi-Modal Health Benefits

**DOI:** 10.3390/antiox14080956

**Published:** 2025-08-04

**Authors:** Mingzhan Xue, Naila Rabbani, Paul J. Thornalley

**Affiliations:** 1 Diabetes Research Center, Qatar Biomedical Research Institute, Hamad Bin Khalifa University, Qatar Foundation, Doha P.O. Box 34110, Qatar; mxue@hbku.edu.qa; 2 Clinical Sciences Research Laboratories, Warwick Medical School, University Hospital, University of Warwick, Clifford Bridge Road, Coventry CV2 2DX, UK; naila.rabbani@glovitality.com; 3 Glovitality Ltd., 167–169 Great Portland Street, London W1W 5PF, UK; 4 College of Health and Life Sciences, Hamad Bin Khalifa University, Qatar Foundation, Doha P.O. Box 34110, Qatar

**Keywords:** methylglyoxal, oxidative stress, lipid peroxidation, ER stress, proteotoxicity, insulin resistance, diabetes, MASLD, aging, resveratrol

## Abstract

A dietary supplement, *trans*-resveratrol and hesperetin (tRES+HESP)—also known as GlucoRegulate—induces increased expression of glyoxalase 1 (Glo1) by activation of transcription factor Nrf2, countering accumulation of the reactive dicarbonyl glycating agent, methylglyoxal. tRES+HESP corrected insulin resistance and decreased fasting and postprandial plasma glucose and low-grade inflammation in overweight and obese subjects in a clinical trial. The aim of this study was to explore, for the first time, health-beneficial gene expression other than Glo1 induced by tRES+HESP in human endothelial cells and fibroblasts in primary culture and HepG2 hepatoma cell line and activity of *cis*-resveratrol (cRES) as a Glo1 inducer. We measured antioxidant response element-linked gene expression in these cells in response to 5 µM tRES+HESP by the NanoString method. tRES+HESP increases gene expression linked to the prevention of dicarbonyl stress, lipid peroxidation, oxidative stress, proteotoxicity and hyperglycemia-linked glycolytic overload. Downstream benefits were improved regulation of glucose and lipid metabolism and decreased inflammation, extracellular matrix remodeling and senescence markers. The median effective concentration of tRES was ninefold lower than cRES in the Glo1 inducer luciferase reporter assay. The GlucoRegulate supplement provides a new treatment option for the prevention of type 2 diabetes and metabolic dysfunction–associated steatotic liver disease and supports healthy aging.

## 1. Introduction

**Figure 1:**
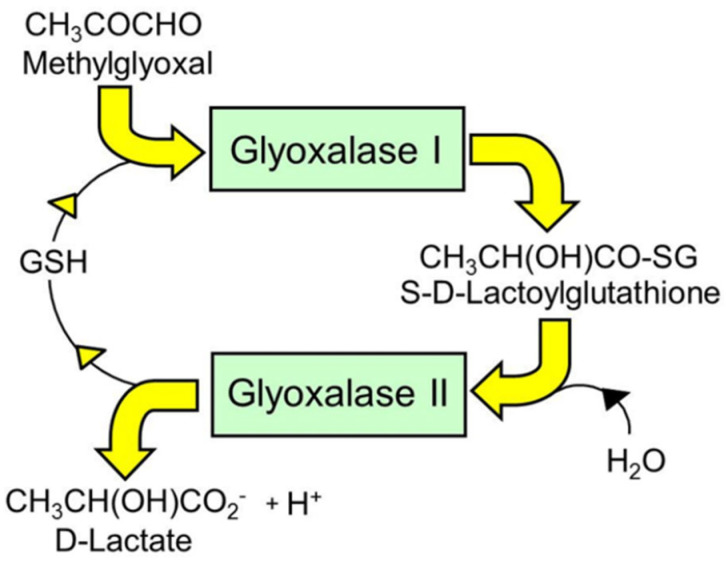
Metabolism of methylglyoxal by the glyoxalase system.

The dietary supplement *trans*-resveratrol and hesperetin (tRES+HESP; also known as GlucoRegulate) is optimized to induce the expression of glyoxalase 1 (Glo1) of the glyoxalase system and decrease the physiological concentration of the reactive metabolite, methylglyoxal (MG) [1]—Figure 1.

Abnormally increased MG, the physiological state of dicarbonyl stress, is found in people who are overweight and those living with obesity, diabetes and renal failure [2]. It is mainly caused by increased formation of MG, formed from an abnormal increase in triosephosphate intermediates of glycolysis, glyceraldehyde-3-phosphate and dihydroxyacetonephosphate, in glycolytic overload and decreased Glo1 resulting from increased cellular proteolysis in hyperglycemia [3,4,5]. Increased MG concentration produces increased glycation of protein and DNA, forming the major physiological advanced glycation end products (AGEs), hydroimidazolone MG-H1 residues of proteins and imidazopurinone MGdG residues of DNA [6,7]. Formation of MG-H1 produces misfolded proteins, which are major physiological ligands activating sensors of the unfolded protein response (UPR) or ER stress: inositol requiring enzyme-1α (IRE1α), protein kinase R-like ER kinase (PERK) and activating transcription factor 6 (ATF6). This increases cellular proteolysis, low grade inflammation and apoptosis [8]. Modification of extracellular matrix (ECM) proteins by MG produces cell detachment from the ECM, anoikis and matrix remodeling [9,10]. Increased DNA glycation produces increased mutation [7,10]. Clinically, these effects are linked to the development of impaired metabolic and vascular health—insulin resistance and chronic vascular complications of diabetes—diabetic kidney disease, diabetic retinopathy, diabetic neuropathy and increased risk of cardiovascular disease, and shortened health span [2]. The best way to counter dicarbonyl stress is to induce increased expression of Glo1, which may be achieved through activation of transcription factor nuclear factor erythroid 2-related factor 2 (Nrf2), exploiting a regulatory antioxidant response element (ARE) in the GLO1 gene [4,10].

We sought to develop and optimize a dietary supplement activator of Nrf2 increasing the expression of Glo1, or Glo1 inducer. tRES+HESP emerged from a screen of dietary bioactive compounds using a luciferase reporter cell line with reporter response linked to the functional ARE of the Glo1 gene, GLO1-ARE [10]. It is the first dietary supplement optimized for expression of a particular gene—a precision dietary supplement. Glo1 inducer activity of tRES+HESP was then validated in human endothelial cells and fibroblasts in primary culture and the HepG2 hepatoma, hepatocyte-like cell line. In a clinical trial—the healthy aging through functional food (HATFF) study—treatment of overweight and obese subjects with tRES+HESP by oral capsule, once daily, containing 90 mg tRES and 120 mg HESP increased expression and activity of Glo1 in peripheral blood mononuclear cells (PBMCs) and decreased the plasma concentration of MG. This thereby validated the expected pharmacology associated with Glo1 inducer treatment. It improved blood glucose control and insulin sensitivity; the placebo was without effect [1]. There were also correlations linking improvements in blood pressure, dyslipidemia and low-grade inflammation [11]. tRES+HESP may therefore find future application in prevention and treatment of disease where insulin resistance and hyperglycemia are risk factors—such as type 2 diabetes mellitus (T2DM) [12,13,14], diabetic kidney disease, diabetic retinopathy and diabetic neuropathy [15,16,17,18,19] and metabolic dysfunction–associated steatotic liver disease (MASLD) [20,21,22]. The chemical structure, main natural sources and an overview of safety evaluation data of tRES, HESP and tRES+HESP are given in Table 1.

At the time of the development of tRES+HESP, it was considered that insulin sensitivity may have been improved by preventing impairment of fibroblast growth factor-21 interaction with receptor cofactor β-Klotho [29]. It was known that MG-driven protein glycation decreased expression of β-Klotho [30]. Since then, the hypothesis of hexokinase-linked glycolytic overload and unscheduled glycolysis has emerged in explanation of hyperglycemia-induced pathogenesis of insulin resistance, beta-cell glucotoxicity, diabetic vascular complications and impaired incretin effect [5,31]. From this, it was proposed that tRES+HESP intervenes at several different levels to improve metabolic health. Firstly, by decreasing cellular MG and formation of MG-derived AGEs, it prevents ER stress and associated increased proteolysis and inflammatory and apoptotic signaling [8]. Indeed, there was decreased inflammatory gene expression in the HATFF with tRES+HESP treatment. Expression of interleukin-8 (IL-8), monocyte chemoattract protein-1 (MCP-1), receptor for advanced glycation endproducts (RAGE) and prostaglandin cyclo-oxygenase-2 (COX-2) was decreased in PBMCs [1]. Secondly, there is a concurrent Nrf2-dependent increase in expression of glucose-6-phosphate dehydrogenase (G6PD) [3]. This increases metabolic flux into the pentosephosphate pathway, increasing formation of NADPH to sustain cellular reduced status and biosynthetic processes. It also decreases the steady-state concentration of glucose-6-phosphate (G6P) and thereby decreases transcriptional activity of G6P-carbohydrate response element binding protein (ChREBP)/Mondo A-Mlx. This is likely crucial for decreasing ChREBP-dependent hepatic insulin resistance and Mondo A-dependent expression of hexokinase-2 (HK2) with consequential decrease in HK2-linked glycolytic overload contributing to peripheral insulin resistance [5]. Prevention of glycolytic overload and ER stress in cells of the incretin effect by tRES+HESP may also counter the impaired incretin effect in obesity and diabetes, enhancing glycemic and appetite control [31]. The proposed mechanisms of health-beneficial responses of GlucoRegulate are summarized in Table 2.

Nrf2 is a vital “master” regulator of cytoprotective responses, regulating the basal and inducible expression of *ca.* 1300 cytoprotective genes [35,36]. Therefore, health beneficial responses other than those linked to increased expression of Glo1 may be available for GlucoRegulate. To explore this, the aim of this study was to assess gene expression of ARE-linked genes and other genes contributing to and reporting on health benefit in response to a clinically relevant concentration of tRES+HESP in vascular, connective tissue and hepatic cell culture models. Herein we report the outcome of studies using human aortal endothelial cells and dermal BJ fibroblasts in primary culture and HepG2 hepatocyte-like cell line treated with 5 µM tRES+HESP and using custom focused gene expression arrays. We also report the importance of the *trans*-isomer compared to the *cis*-isomer of resveratrol in the Glo1 inducer response.

## 2. Materials and Methods

### 2.1. Cell Culture

Primary human aortal endothelial cells (HAEC) were purchased from Lonza (Slough, UK). HAEC cells were grown in proprietary large vessel endothelial cell basal media supplemented with large vessel endothelial cell growth supplement containing hydrocortisone, human epidermal growth factor, human fibroblast growth factor with heparin and in 2% (*v*/*v*) fetal bovine serum (FBS), 25 µg/mL gentamicin and 50 ng/mL amphotericin B. Human dermal foreskin BJ fibroblasts at cumulative population doubling of 22 were purchased from the European Collection of Animal Cell Cultures (Porton Down, UK). They were cultured in Minimum Essential Medium (MEM) medium with 10% FBS and 2 mM glutamine under an atmosphere of 5% CO_2_ in air, 100% humidity and 37 °C. The human HepG2 cell line was cultured as previously described [10]. Tissue culture materials, medium MEM and L-glutamine were from Invitrogen (Paisley, UK), and fetal bovine serum was from Biosera (Ringmer, UK). Human large vessel endothelial cell growth medium packages were from Caltag Medsystems (Buckingham, UK). tRES (≥99% purity) and cRES (≥99% purity) were purchased from Merck (Poole, UK) and HESP (≥98% purity) from Cambridge Bioscience (Cambridge, UK).

### 2.2. ARE-Linked Gene Expression and Other Cell Metabolism and Vitality Marker Gene Expression by Digital mRNA Profiling

HAECs, BJ fibroblasts and HepG2 cells (5 × 10^5^ cells/well) were seeded on 6-well plates in MCDB-131 medium and cultured overnight at 37 °C under 5% CO_2_/air. Cells were treated with and without 5 μM tRES+HESP (5 μM tRES and 5 μM HESP combined) or vehicle (0.002% DMSO) and cultured further for up to 48 h (HAECs and HepG2 cells) or 72 h (BJ fibroblasts). At the desired time point, cells were washed twice with ice-cold phosphate-buffered saline, and total RNA was extracted using RNeasy Mini Kit (Qiagen). Total RNA (600–800 ng) was analyzed for mRNA copy number of target genes by the NanoString nCounter Gene Expression method [37] with a custom codeset of test genes and three reference genes (β-actin, clathrin heavy chain and β-glucuronidase) included in the custom array design (outsourced to NanoString, Seattle, WA, USA).

### 2.3. Glyoxalase 1 Inducer Response Using GLO1-ARE and Related Mutant Stable Transfectant Reporter Cells Lines

Stable transfectant luciferase reporter cell lines with ARE transcriptional regulatory elements were developed from human HepG2 cells, as described for quinone reductase ARE [38], incorporating regulatory elements: GLO1-ARE or functionally inactive mutant as negative control (described as ARE-1 and ARE1m in our previous work) [10]. Stable transfectant cell lines were incubated with tRES and cRES, 0.625–40 μM, or vehicle (0.002% DMSO for 6 h). Luciferase activity was then determined in cell lysates, correcting for blank response and normalized to the highest effect (100%) achieved with 10 μM tRES [10]. Data of normalized responses for varied RES concentrations were fitted by nonlinear regression to the equation E = E_max_ × [RES]^n^/(EC_50_^n^ + [RES]^n^), solving for E_max_, EC_50_, and n (Hill coefficient) using the ENZFITTER program (Biosoft, Cambridge, UK).

### 2.4. Statistical Analyses

Data are mean ± SD of 3 independent biological replicates. Significance of difference was assessed by Student’s *t*-test (2 groups). Data shown for time courses have been normalized at each time point to the untreated control. Only treatment responses are shown for clarity. Statistical calculations were performed in Microsoft 365 Excel worksheets.

## 3. Results

### 3.1. Changes in ARE-Linked Gene Expression Induced by GlucoRegulate in Human Aortal Endothelial Cells In Vitro

**Figure 2:**
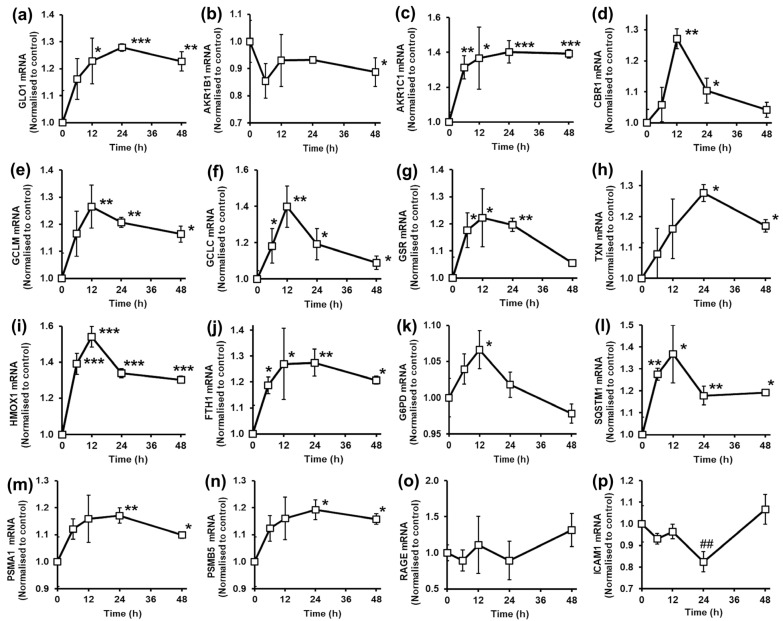
Effect of glyoxalase 1 inducer, *trans*-resveratrol and hesperetin combination on gene expression in human aortal endothelial cells in primary culture. mRNA copy number, normalized to control (unstimulated cells). Genes: (**a**) GLO1. (**b**) AKR1B1. (**c**) AKR1C1. (**d**) CBR1. (**e**) GCLM. (**f**) GCLC. (**g**) GSR. (**h**) TXN. (**i**) HMOX1. (**j**) FTH1. (**k**) G6PD. (**l**) SQSTM1. (**m**) PSMA1. (**n**) PSMB5. (**o**) RAGE. (**p**) ICAM1. HAECs were incubated with and without 5 µM tRES+HESP. Data are mean ± SD (*n* = 3). Significance: *, ** and ***, *p* < 0.05, *p* < 0.01 and *p* < 0.001 for mRNA increases with respect to unstimulated control and ##, *p* < 0.01 for mRNA decrease with respect to unstimulated control; Student’s *t*-test.

When HAECs were incubated with 5 µM tRES+HESP, Glo1 mRNA was increased to a maximum at 24 h and thereafter gradually decreased (Figure 2a).

Other carbonyl compound metabolizing ARE-linked genes expressed in endothelial cells are aldose reductase (AKR1B1), 3*α*(20*α*)-hydroxysteroid dehydrogenase (AKR1C1) and carbonyl reductase1 (CBR1). Aldose reductase has low expression in HAECs and did not contribute to MG metabolism. Indeed, MG reductase activity was undetectable in HAECs in our previous studies [3]. Aldose reductase mRNA levels were little changed by tRES+HESP (Figure 2b). AKR1C1 metabolizes damaging 4-hydroxy-2-nonenal (4-HNE) and other α,β-unsaturated aldehydes formed during lipid peroxidation to innocuous products [39]. tRES+HESP increased expression of AKR1C1 over the initial 12 h, which was sustained to 48 h (Figure 2c). CBR1 catalyzes the reduction of adducts formed spontaneously with reduced glutathione (GSH) and reactive aldehydes derived from lipid peroxidation [40]. tRES+HESP increased mRNA levels sharply up to 12 h, which then decreased thereafter to approach basal levels by 48 h (Figure 2d). So, tRES+HESP increases endogenous protection against substrates of Glo1, glyoxal and MG and other reactive aldehydes formed from lipid peroxidation.

Nrf2 is also an important regulator of gene expressions of glutathione synthesis and metabolism. γ-Glutamylcysteine ligase (GCL) modulatory and catalytic subunits, GLM and GCLC, mRNA were both increased by tRES+HESP, maximizing at 12 h and decreasing thereafter (Figure 2e,f). There was a similar increase also in glutathione reductase (GSR) mRNA levels induced by tRES+HESP (Figure 2g). A further thiol maintaining reduced status of proteins in cells is the small 11.7 kDa protein with two vicinal cysteinyl thiols, thioredoxin (TXN). Treatment of HAECs with tRES+HESP increased thioredoxin mRNA levels, maximizing after 24 h and decreasing thereafter (Figure 2h).

Nrf2 also regulates the expression of genes involved in heme catabolism and iron storage: heme oxygenase-1 (HMOX1) and ferritin (FTH1). Treatment of HAECs with tRES+HESP increased mRNA levels of HMOX1, maximizing at 12 h and slowly decreasing thereafter (Figure 2i). mRNA levels of FTH1 also increased at 12–24 h and then slowly decreased (Figure 2j).

Increased expression of G6PD in response to tRES+HESP in HAECs is implicated in correction glycolytic overload and cell dysfunction in hyperglycemia [3]. mRNA levels of G6PD increased rapidly with tRES+HESP treatment, maximizing at 12 h and decreasing to basal levels by 24 h (Figure 2k). SQSTM1 is an ARE-regulated gene expressing p62 protein, which provides a scaffold to direct protein substrates to autophagy. p62 also interacts with Keap1 to release Nrf2 and stimulate expression of p62 and ARE-linked genes—reviewed in [41]. In HAECs, treatment with tRES+HESP increased SQSTM1 mRNA levels rapidly to maximize at 6–12 h. SQSTM1 mRNA levels decreased slightly by 24 h thereafter, with a sustained continued modest increased to 48 h (Figure 2l). Activation of Nrf2 also increases expression of subunits of the proteasome [42]. Treatment of HAECs with tRES+HESP increased mRNA levels of proteasome subunit alpha type-1 (PSMA1) and subunit beta type-5 (PSMB5), increasing in the initial 24 h and decreasing slowly thereafter (Figure 2m,n). Regarding downstream functional responses, we assessed the expression of RAGE and ICAM1. In HAECs, the level of RAGE mRNA was unchanged by treatment with tRES+HESP, whereas ICAM1 mRNA was decreased at 24 h and returned to basal levels at 48 h (Figure 2o,p). Nevertheless, RAGE and ICAM1 were decreased in HAECs treated with tRES+HESP at the protein level—which may have been linked to improved proteostasis. In the HATFF clinical study, RAGE mRNA in PBMCs and ICAM1 protein in plasma were decreased with tRES+HESP treatment [1].

### 3.2. Changes in ARE-Linked Gene Expression Induced by GlucoRegulate in Human BJ Fibroblasts In Vitro

**Figure 3:**
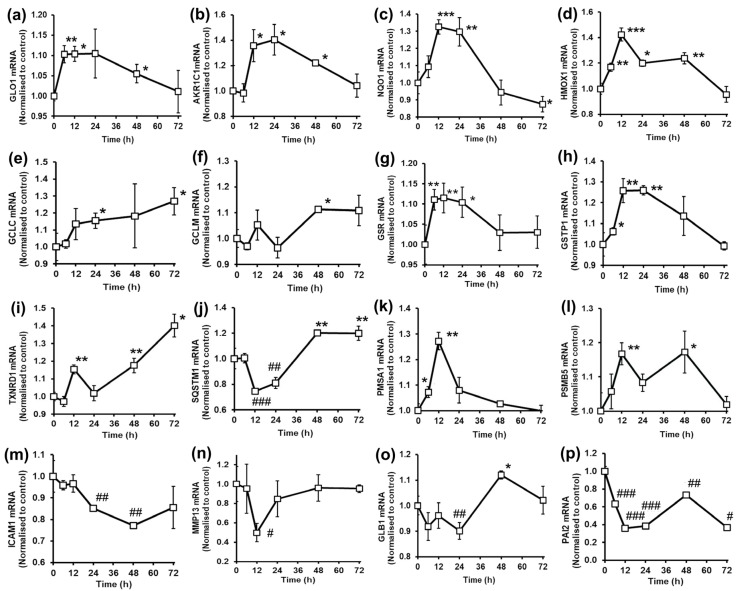
Effect of glyoxalase 1 inducer, *trans*-resveratrol and hesperetin combination on gene expression in human BJ fibroblasts in primary culture. mRNA copy number, normalized to control (unstimulated cells). Genes: (**a**) GLO1. (**b**) AKR1C1. (**c**) NQO1. (**d**) HMOX1. (**e**) GCLC. (**f**) GCLM. (**g**) GSR. (**h**) GSTP1. (**i**) TXNRD1. (**j**) SQSMT1. (**k**) PMSA1. (**l**) PSMB5. (**m**) ICAM1. (**n**) MMP13. (**o**) GLB1. (**p**) PAI2. BJ fibroblasts were incubated with and without 5 µM tRES+HESP. Data are mean ± SD (n = 3). Significance: *, ** and ***, *p* < 0.05, *p* < 0.01 and *p* < 0.001 for mRNA increases with respect to unstimulated control and #, ##, and ###, *p* < 0.05, *p* < 0.01 and *p* < 0.001 for mRNA decreases with respect to unstimulated control; Student’s *t*-test.

In human BJ fibroblasts, treatment with tRES+HESP increased expression of GLO1 and AKR1C1, with mRNA levels optimizing at 6–24 h for GLO1 and 12–24 h for AKR1C1 (Figure 3a,b).

Quinone reductase (NQO1) is an ARE-related gene where increase in expression is often used as a marker of Nrf2 activation [43]. tRES+HESP treatment produced a rapid increase in levels of NQO1 mRNA, maximizing at 12–24 h and thereafter decreasing to basal levels at 48 h and below basal levels at 72 h (Figure 3c). mRNA levels of other ARE-regulated genes typical of Nrf2 activation were increased: HMOX1, GCLC, GCLM and GSR (Figure 3d–g). Glutathione S-transferases (GSTs) are ARE-regulated genes, and GSTP1 is the major isoform expressed in human skin fibroblasts [44]. Treatment of BJ fibroblasts with tRES+HESP increased levels of GSTP1 mRNA rapidly, maximizing at 12–24 h and decreasing to basal levels thereafter (Figure 3h). Cytosolic thioredoxin reductase-1 (TXNRD1) is an ARE-linked gene, which reduces thioredoxin and thereby provides reducing equivalents to ribonucleotide reductase, peroxiredoxins and methionine sulfoxide reductases [45]. The level of TXNRD1 mRNA of BJ fibroblasts was increased by tRES+HESP treatment, maximizing after 72 h (Figure 3i). tRES+HESP treatment of BJ fibroblasts produced an unusual response in SQSTM1 mRNA levels, producing a decrease at 12–24 h and increasing above basal levels thereafter at 48 and 72 h (Figure 3j). It also produced increased levels of mRNA of proteasome subunits PMSA1 and PSMB5 (Figure 3k,l). Assessing expression of functional biomarkers, there were decreases in mRNA levels of the inflammatory biomarker, ICAM1, and extracellular matrix remodeling biomarker, matrix metalloproteinase-13 (MMP13) – Figure 3m. There was an undulating level of β-galactosidase (GLB1) mRNA and a continual decrease of plasminogen activator inhibitor-2 (PAI2) (Figure 3o,p). These are both biomarkers of cell senescence [46,47].

### 3.3. Changes in ARE-Linked Gene Expression Induced by GlucoRegulate in Human HepG2 Cells In Vitro

**Figure 4:**
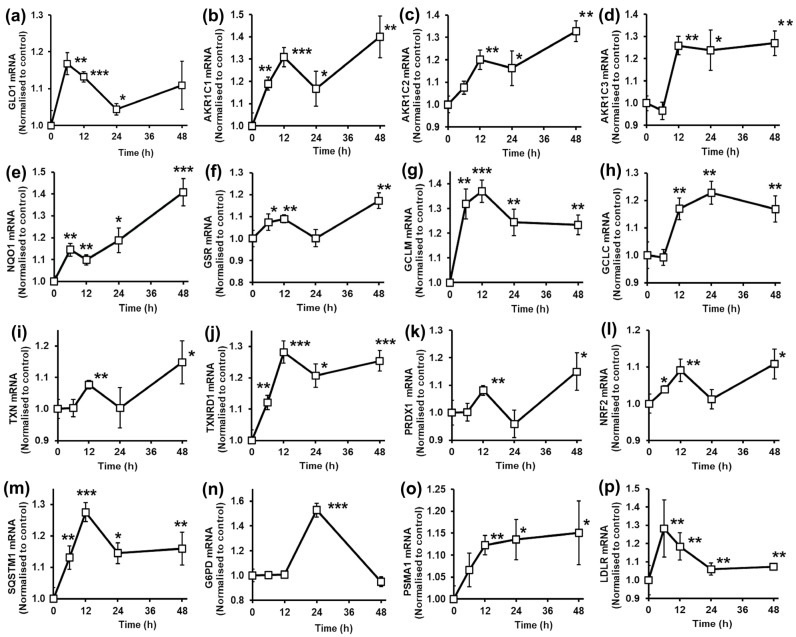
Effect of glyoxalase 1 inducer, *trans*-resveratrol and hesperetin combination on gene expression in human HepG2 cells in vitro. mRNA copy number, normalized to control (unstimulated cells). Genes: (**a**) GLO1. (**b**) AKR1C1. (**c**) AKR1C2. (**d**) AKR1C3. (**e**) NQO1. (**f**) GSR. (**g**) GCLM. (**h**) GCLC. (**i**) TXN. (**j**) TXNRD1. (**k**) PRDX1. (**l**) NRF2. (**m**) SQSTM1. (**n**) G6PD. (**o**) PSMA1. (**p**) LDLR. HepG2 cells were incubated with and without 5 µM tRES+HESP. Data are mean ± SD (n = 3). Significance: *, ** and ***, *p* < 0.05, *p* < 0.01 and *p* < 0.001 with respect to unstimulated control; Student’s *t*-test.

HepG2 cells were studied as an in vitro model of human hepatocytes. When HepG2 cells were treated with 5 µM tRES+HESP, the levels of Glo1 and aldoketo reductases AKR1C1, AKR1C2 and AKR1C3 were increased (Figure 4a–d).

There is a suggestion of biphasic response: mRNA increasing to a temporary maximum at 6–12 h and thereafter a further second phase of increase to 48 h. This was also found for NQO1 and GSR (Figure 4e,f). mRNA levels of GCLM and GCLC were also increased in response to tRES+HESP with an initial increase at 6–24 h only and slow decline thereafter (Figure 4g,h). Other ARE-regulated genes, TXN, TXNRD1, peroxiredoxin-1 (PRDX1) and Nrf2, also showed a biphasic increase in mRNA levels in response to tRES+HESP (Figure 4i–l). SQSTM1 mRNA levels were increased rapidly in the initial 12 h in response to tRES+HESP, and, as p62 protein is an activator of Nrf2 [48], increased p62 protein following increase in mRNA may explain the second phase of increased mRNA of several ARE-linked genes (Figure 4m). Increased mRNA of G6PD appeared to occur only after a delay of 12 h (Figure 4n), whereas increased expression of proteasome subunits PSMA1 occurred from 12 to 48 h (Figure 4o). In HepG2 cells, treatment with tRES+HESP induced a rapid increase in mRNA levels of the LDL receptor (LDLR), which later decreased but remained above basal levels to 48 h post-treatment (Figure 4p).

### 3.4. Comparison of Glyoxalase 1 Inducer Activity of Geometric Isomers of trans-Resveratrol and cis-Resveratrol

**Figure 5:**
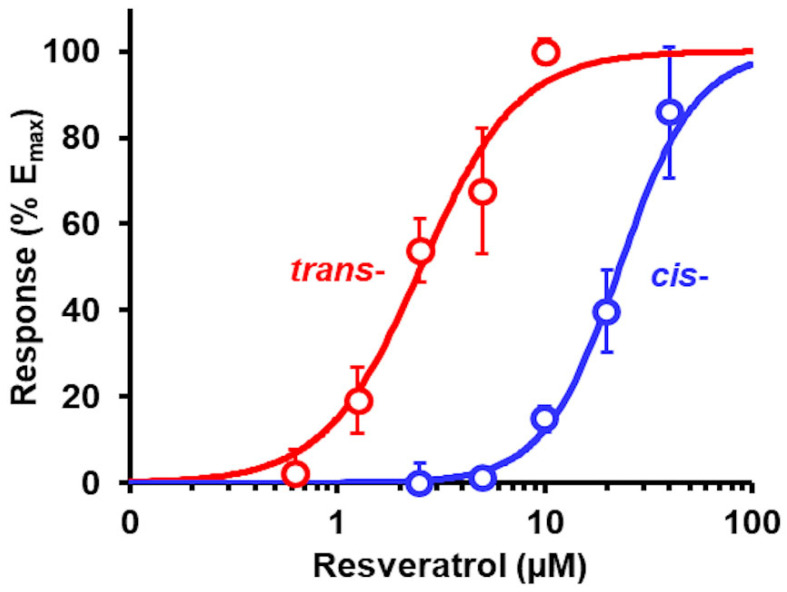
Induction of Glo1 expression by tRES and cRES. GLO1-ARE transcriptional response reporter assay. Data are mean ± SD (*n* = 3) for 5 concentrations. Nonlinear regression curves: tRES (red curve), E (%) = 100 × [tRES]^1.99^/(2.52^1.99^ + [tRES]^1.99^); and cRES (red curve), E (%) = 100 × [cRES]^2.36^/(23.0^2.36^+ [cRES]^2.36^).

The geometric isomers of resveratrol, *trans*- and *cis-*, are often found together in natural sources of resveratrol. It is therefore of interest if this isomerism affects the Glo1 inducer activity. We assessed this in the GLO1-ARE luciferase reporter assay. We found that the median effective concentration EC_50_ value of tRES was ninefold lower than that of cRES. EC_50_ for Glo1 induction: tRES, EC_50_ = 2.52 ± 0.19 µM, n = 1.99 ± 0.26; and cRES, EC_50_ = 23.0 ± 1.16 µM, n = 2.36 ± 0.27 (N = 15)—Figure 5.

## 4. Discussion

### 4.1. Nrf2-Mediated Health Beneficial Gene Expression Changes of GlucoRegulate in HAECs, BJ Fibroblasts and HepG2 Cells

The present study focused on ARE-linked gene expression in HAECs, BJ fibroblasts and HepG2 cells and the effect of treatment with Glo1 inducer, 5 µM GlucoRegulate [1]. The overall interpretation of the time course plots of change in target gene mRNA copy number is as follows: tRES and HESP enter the cells and activate Nrf2 to produce the transactivational response; this increases or decreases the rate of transcription of the target gene, producing an initial increase or decrease in mRNA copy number. Meanwhile, tRES and HESP are slowly metabolised to glucuronide and sulfate conjugates, which have decreased Nrf2 activation activity and are also exported from the cells. At this stage, the rate of transcription of the target gene returns to basal levels, and the mRNA copy number of the target genes returns to baseline levels, the rate of which depends on the half-life of the particular mRNA. There may be a biphasic, second wave of mRNA increase where increased SQSTM1 expression in the initial phase leads to increased p62 protein, which stimulates a second wave of ARE-linked gene expression [48].

mRNA levels of genes assessed often showed increases maximizing at 6–24 h and thereafter slowly decreasing to 48 h and 72 h post-treatment. In previous studies, 10 µM and 15 µM tRES had been found to increase GCLC and HMOX1 expression [49,50] and 10–30 µM HESP had been found to increase GCLM, HMOX1 and NQO1 expression [51]. The synergism of tRES and HESP in combination, active ingredients of GlucoRegulate, in the activation of Nrf2 for increased ARE-linked gene expression herein is novel, where together, lower concentrations of tRES and HESP were effective [1]. In this study, we report changes in ARE-linked gene expression other than Glo1 in response to tRES+HESP, which likely also contributes to the clinical health-beneficial response.

In HAECs, we found that GlucoRegulate increased expression of AKR1C1 and CBR1, which, through metabolism of 4-HNE, other α,β-unsaturated aldehydes and GSH adducts thereof, likely provides enhanced protection against damaging reactive carbonyl metabolites derived from lipid peroxidation [39,40]. GlucoRegulate also enhanced the antioxidant reserve of HAECs by increasing the expression of GCL subunits, GLCM and GCLC, TXN, HMOX1, FTH1 and G6PD. This may increase the cellular capability for synthesis of GSH and TXN, handling of heme iron metabolism and storage and increased NADPH to support GSR and TXNRD activity. Although treatment with GlucoRegulate did not increase the concentration of GSH in HAECs [1], it may provide increased GSH synthesis activity such that GSH levels may be preserved better when HAECs are under oxidative challenge—such as in high glucose concentration [3,32]. Increased G6PD also had a critical role in protection of HAECs from glycolytic overload in model hyperglycemia where high cytosolic glucose concentration stabilizes HK2 to proteolysis and increases flux of glucose metabolism into glycolysis without increase in other glycolytic enzyme expression and activity - unscheduled glycolysis [5]. This is corrected by increased G6PD through decreasing G6P/Mondo A/Mlx-dependent expression of HK2, correcting HAEC dysfunction in high glucose concentration [3]. GlucoRegulate also increased expression of p62 and proteasomal subunits, increasing surveillance and quality of proteostasis and thereby enhancing protection against proteotoxicity. Regarding downstream functional responses, there was no change in mRNA levels of RAGE in HAECs, whereas at the protein level, RAGE, ICAM1 and E-selectin were all decreased with GlucoRegulate treatment [1]. This may be an anti-inflammatory effect related to improved proteostasis. Thus, HAECs treated with GlucoRegulate have increased protection against dicarbonyl stress and activation of the UPR, lipid peroxidation, oxidative stress, hyperglycemia-linked glycolytic overload, proteotoxicity and low-grade inflammation.

In BJ fibroblasts, GlucoRegulate again afforded protection against dicarbonyl stress and lipid peroxidation through increased expression of Glo1 and AKR1C1. Cytoprotection against oxidative stress was indicated by increased expression of NQO1, GCLC, GCLM, GSR, HMOX1, GSTP1 and TXNRD1. Treatment of BJ fibroblasts with 5 µM GlucoRegulate increased the cellular concentration of GSH by 43%, whereas treatment with tRES and HESP individually was ineffective [1]. This increases the antioxidant reserve and also further increases in situ activity of Glo1 [2]. Resistance to proteotoxicity was indicated by increased expression of SQSTM1 and proteasome subunits. For downstream functional changes, there was decreased ICAM1, MMP13 and SERPINB2. This suggested that GlucoRegulate afforded fibroblast resistance to dicarbonyl stress, lipid peroxidation, oxidative stress and proteotoxicity. The downstream benefits were as follows: decreased low grade inflammation—as indicated by decreased ICAM1 expression and decreased RAGE at the protein level [1]; decreased ECM remodeling by decreased expression of MMP13—a key factor in ECM proteolysis in the progression of osteoarthritis [52]; and decreased biomarkers of cellular senescence—temporary decreases in GLB1 and SERPINB2 [46,47]. Decrease of fibroblast replicative senescence by tRES has been found previously; treatment of MRC5 fibroblasts with 5 µM tRES produced a small delay in replicative senescence [53]. We also found that similar treatment with 5 µM HESP produced a small delay in replicative senescence (F. Hariton and P.J. Thornalley, unpublished observations). Together, therefore, 5 µM tRES+HESP may delay fibroblast replicative senescence. This remains to be investigated. At higher concentrations (50 µM and 100 µM), tRES induced premature replicative senescence [54]. This is likely due to tRES engaging with receptors other than those leading to the activation of Nrf2 at high concentrations—see below. In other studies, we found that GlucoRegulate prevented HK2-linked glycolytic overload and metabolic dysfunction of fibroblasts in model hyperglycemia [32]. Suppression of glycolysis in fibroblasts by GlucoRegulate may also contribute to decreased replicate senescence, as increased glycolysis was a key driver to the approach to senescence in fibroblasts [55].

In BJ fibroblasts, mRNA levels of NQO1 at 72 h and SQSTM1 at 12 h and 24 h were decreased with GlucoRegulate treatment. This may be linked to crosstalk between Nrf2 and the aryl hydrocarbon receptor (AhR)—the latter antagonized by low concentrations of tRES and HESP [56,57]—see below. Expression of NQO1 may be induced by activated AhR interacting with Nrf2 [58] and expression of SQSTM1 by AhR via protein phosphatase 2 regulatory subunit-Bdelta (PPP2R2D) [59]. At the time points when target gene mRNA levels are decreased, the antagonist of AhR-driven expression may be dominant; and when increased, the Nrf2-linked mRNA increase is dominant.

In HepG2 cells, GlucoRegulate again afforded protection against dicarbonyl stress and lipid peroxidation through increased expression of Glo1, AKR1C1, AKR1C2 and AKR1C3. Cytoprotection against oxidative stress was indicated by increased expression of NQO1, GCLM, GCLC, GSR. TXN, TXNRD1, PRDX1, Nrf2 and G6PD. Treatment of HepG2 cells with 5 µM GlucoRegulate increased the cellular concentration of GSH by 32%, whereas treatment with tRES and HESP individually was ineffective [1]. This increases the antioxidant reserve and also further increases in situ activity of Glo1, as found in BJ fibroblasts—see above. Resistance to proteotoxicity was suggested by increased expression of SQSTM1 and proteasome subunit PSMA1. Later increased p62 protein likely activating Nrf2 [41] may account for the second phase of increased ARE-linked gene expression.

In HepG2 cells, GlucoRegulate increased the expression of LDLR, although this is not an ARE-regulated gene [60]. tRES alone increased LDLR expression at the mRNA and protein levels, albeit less effectively than when combined with HESP [61]. tRES-induced increased expression of LDLR is thought to occur via increased activity of the sterol regulatory element-binding protein-2 (SREBP-2) [62]. It is uncertain how this occurs, but it may be mediated by antagonism of tRES at the AhR. Active AhR otherwise decreases expression of SREBP-2 [63]. If replicated in human hepatocytes in situ in the liver, GlucoRegulate treatment may facilitate hepatic uptake of LDL and support improved clinical lipid metabolism. Although plasma markers of lipidemia were not changed by treatment with tRES+HESP in the HATFF study, correlation between MG-related variables and lipoprotein levels suggest that GlucoRegulate treatment may weakly support improvement of dyslipidemia [11].

Overall, this suggests that GlucoRegulate afforded HepG2 hepatocyte-like cells protection against dicarbonyl stress, lipid peroxidation, oxidative stress, proteotoxicity, glycolytic overload and hypercholesterolemia. If similar responses are found in human hepatocytes in vivo, this may contribute to improved hepatic glucose metabolism, correction of hepatic insulin resistance and improved lipid metabolism.

### 4.2. Glyoxalase 1 Inducer Activity of trans- and cis-Resveratrol

Our studies indicate that tRES is a markedly more potent Glo1 inducer than cRES, with an EC_50_ value *ca.* ninefold lower than that of cRES; 2.5 µM vs 23 µM. Peak plasma concentrations of tRES are <5 µM [64]. Peak plasma concentrations of cRES clinically are likely even lower than for tRES, as cRES has more rapid presystemic metabolism [65]. It is likely, therefore, that Nrf2-related health responses are not available clinically with cRES. It appears that GlucoRegulate was appropriately formulated with the tRES isomer. cRES is often found to be less effective in biological responses than tRES—antiproliferative effect [66], LDLR increase [61,67] and others; tRES and cRES have similar chemical free radical scavanging activity [68]. For health beneficial responses found herein and for clinical effectiveness in vivo, tRES is a better supplement component than cRES.

### 4.3. Pharmacological Targets of trans-Resveratrol and Hesperetin Likely Involved in the Health Beneficial Responses

Considering the mechanism of the pharmacological responses to tRES+HESP supporting the health benefits found clinically, responses induced by clinically achievable concentrations of tRES and HESP are relevant. Can concentrations of tRES and HESP similar to the EC_50_ concentrations of the Glo1 inducer response, 1.5 µM and 0.6 µM, respectively [1], be achieved clinically? We were confident of achieving effective plasma concentrations of HESP as the peak plasma concentration of *ca.* 6 µM was found with 150 mg HESP—a highly tolerated and safe dose similar to that used in the HATFF study. We remained concerned if an effective concentration of tRES could be achieved. tRES is well tolerated clinically at doses of <1 g [25]. tRES has poor bioavailability and also decreasing absorption as the dose increases [69]. With an oral dose of 25 mg tRES, the peak plasma concentration of tRES, C_max_, was <0.02 µM and the absorption ≥70% in healthy participants [70]. With an oral dose of 75 mg tRES, the plasma concentration of tRES was 0.5 ± 0.8 µM (measured one day after dosing, and therefore C_max_ was likely up to 10-fold higher) [64,71]. Dose escalation studies in healthy human subjects with 0.5–5 g tRES gave estimates of C_max_ values of *ca.* 0.2–2.5 µM with total metabolites excreted in the initial 24 h representing 23–10% of the administered dose [64]. Low bioavailability of tRES is due to efficient pre-systemic metabolism of tRES by glucuronosyltransferases to 3-O-glucuronide and 4′-O-glucuronide in intestinal epithelial cells [72] and post-absorption metabolism in the liver [73]. There is also metabolism to resveratrol 3-O-sulfate [64,70]. Therefore, in clinical translation, we could expect to achieve tRES plasma concentrations no higher than the low micromolar range [74]. We evaluated the Glo1 inducer response of tRES in the presence of clinically achievable 5 µM HESP and found the EC_50_ of tRES decreased to 1.5 µM whilst maintaining the high maximal effect E_max_ of tRES (*ca.* fourfold higher than the E_max_ of HESP alone) [1]. HESP also inhibits intestinal glucuronosyltransferases [75], and therefore co-administration of HESP with tRES clinically temporarily inhibits pre-systemic glucuronidation of both tRES and HESP itself, providing for increased bioavailability of tRES [4]. Therefore, in the HATFF study with dosing of 90 mg tRES and 120 mg HESP, we could expect to achieve a clinically translatable Glo1 inducer response. This was confirmed in the HATFF study, where tRES+HESP produced markedly better improvement in metabolic health than has been achieved by similar doses of tRES alone [1]. The benefit of the tRES+HESP combination was expected due to synergism of tRES and HESP in the activation of Nrf2 and improved bioavailability of tRES [4].

There remains consideration of how tRES+HESP activates Nrf2. Prospective receptors listed by increasing concentration of tRES or HESP required for effectiveness are given in Table 3.

**Figure 6:**
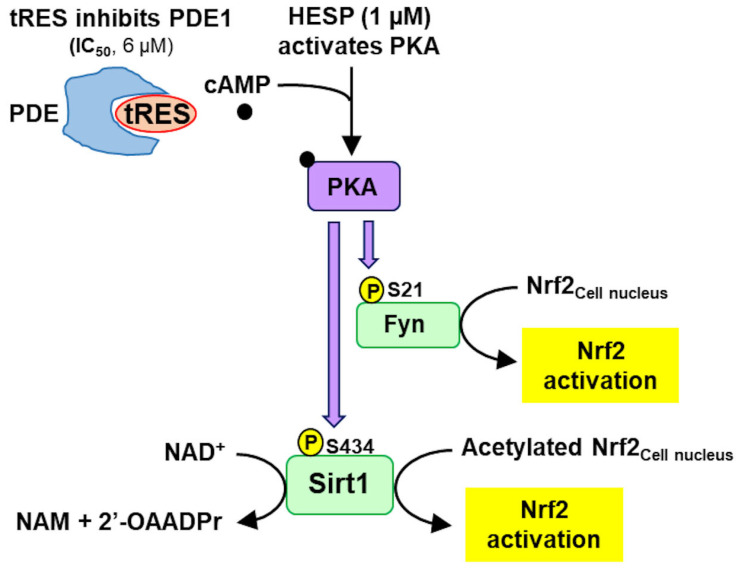
Pharmacological synergism of *trans*-resveratrol and hesperetin to activate sirtuin-1 and fyn kinase. See text. Abbreviations: PDE, phosphodiesterase; NAM, nicotinamide; 2′-OAADPr, 3′-O-acetyl-ADP-ribose; PKA, protein kinase A; Fyn, Fyn kinase; P, phosphorylation.

The mechanism of activation of Nrf2 remains incompletely understood. Nrf2 binds to the protein Keap1 in the cytosol. This created complex fragments, and Nrf2 protein undergoes translocational oscillations between the cell cytosol and nucleus, continually sensing and adjusting cytoprotective gene expression to counter cell stresses—working like a wireless sensor. The frequency of the translocational oscillations increases in response to Nrf2 activators, leading to a concomitant increase in the level of functionally active Nrf2 in the nucleus and ARE-linked transactivational responses [38]. Nrf2 translocation into the nucleus was mediated by phosphorylation by casein kinase-2; once in the nucleus, Nrf2 is inactivated by acetylation and returns to the cytoplasm after phosphorylation by fyn kinase, where it was reactivated by de-acetylation and dephosphorylation [38]. Activation of Nrf2 may occur by small molecules modifying Keap1 and facilitating liberation of Nrf2 from complexation with Keap1 for translocation to the nucleus. This is likely the mechanism of activation of Nrf2 by sulforaphane under dose-limiting conditions [38]. Synergism of tRES and HESP in activation of Nrf2 was consistent with tRES slowing the inactivation of Nrf2 in the nucleus by increasing activity of deacetylase, sirtuin-1, and HESP increasing the rate of expulsion of Nrf2 from the nucleus and re-activation by activation of fyn kinase [1,4]. At low concentrations, tRES is an inhibitor of phosphodiesterase-1 (PDE1), which increases cellular cAMP concentration and *in situ* activity of PKA [76]. Increased protein kinase A (PKA) activity activates sirtuin-1 by phosphorylation on serine-434 [85]; tRES did not activate sirtuin-1 directly at clinical concentrations [86]. HESP is an activator of PKA at 1 µM [82]. tRES and HESP thereby synergize at cAMP/PKA to activate sirtuin-1 to maintain active Nrf2 in the cell nucleus and activate Fyn kinase to expel inactivate Nrf2 from the nucleus for reactivation in the cytosol [87]. Thereby, potent activation of Nrf2 and ARE-linked gene expression response occurs [4]—Figure 6.

From concentration-response considerations, it is also clear that the inhibition of the AhR by tRES and HESP may also be involved (Table 3). Mice deficient in AhR expression were resistant to high fat diet (HFD)-induced obesity and had increased gene expression for energy expenditure through increased brown fat UCP1-linked thermogenesis and muscle fatty acid β-oxidation. AhR deficiency protected against HFD-induced obesity, hepatic steatosis, insulin resistance and inflammation [77]. If antagonism by tRES and HESP at AhR produces the same effects clinically, this may have contributed to health-beneficial effects of tRES+HESP in the HATFF study.

Other effects of tRES likely do not achieve clinical translation because the effective concentration is below the peak plasma concentration of tRES. These are inhibition of mitochondrial F0F1-ATPase/ATP synthase and activation of AMP kinase (AMPK), inhibition of phosphoinositide 3-kinase (PI3K), activation of exchange protein directly activated by cAMP-1 (Epac1) and mitochondrial permeability transition pore-linked apoptosis (Table 3). Inhibition of ATP synthase may have a role in the cytotoxicity of tRES [78]. Activation of AMPK contributed to the health benefits of high-dose tRES in mice [88,89], but no increase in AMPK activity was found with tRES treatment clinically [71]. Inhibition of PI3K would likely induce insulin resistance [80] rather than correct it as found in the HATFF study [1]. Where high, supraphysiological concentrations of tRES are used for in vitro studies, adverse effects may be found; for example, premature senescence of fibroblasts with 50 µM and 100 µM tRES [54]. Apoptotic activity of tRES was of interest in anticancer studies, but clinical utility became uncertain when treatment with very high dose tRES (5 g per day) was suspected to be linked to patient fatality [90]. A 26-week study in mice of very high dose tRES (1g/kg/day) produced renal toxicity [91] for which the equivalent dose in adult human subjects is *ca.* 5.7 g per day [92]. Minor clinical adverse effects generally begin to present clinically at doses of ≥1g tRES (diarrhea or other gastrointestinal symptoms) with severe adverse effects expected at higher doses [25].

High concentrations of HESP were found to inhibit PDE-4 and induce Bax-linked mitochondrial pathway of apoptosis, but such concentrations are likely unachievable clinically (Table 3). Therefore, we suggest that health benefits of tRES with HESP may be available at plasma concentrations of 1–5 µM tRES and HESP.

Finally, combining tRES and HESP together in GlucoRegulate supplement produced clinical translation of a multi-modal health beneficial response: prevention of dicarbonyl stress, ER stress, glycolytic overload and proteotoxicity, antioxidant, anti-inflammatory and potent insulin sensitizer activity in vivo (Table 3). There appears to be a plasma concentration range of *ca.* 1–5 µM tRES+HESP, a clinical pharmacological “sweet spot,” where improvements in metabolic and vascular health are available. Strategies to improve bioavailability of tRES to achieve higher plasma concentrations may be counterproductive through engagement of receptors with adverse effects. Efficient metabolism and decreased efficiency of uptake of tRES at high doses likely protect against achieving toxic concentrations and adverse effects clinically up to doses of 1 g. In review of safety of tRES and assessment of toxicity of HESP, there was greater risk of adverse effects of high concentrations tRES than of high concentrations of HESP [25,28]. A dose of 150 mg tRES taken chronically was considered safe [25]. The synergistic combination of tRES with HESP in GlucoRegulate allows for high clinical effectiveness at relatively low doses (90 mg tRES and 120 mg HESP) with high safety margin and tolerability.

The major limitations of this study are that it is in vitro experimentation with assessment of relative mRNA copy number changes in human primary cell cultures and hepatocyte-like HepG2 cell line and assessment of Nrf2-mediated expression of Glo1 using stable transfectant GLO1-ARE luciferase reporter cells lines. These cell culture models have, however, provided valuable insight into health-beneficial responses of GlucoRegulate in previous studies [1]. Further studies are required to validate the gene expression responses reported herein at the protein level in the cell culture models used herein and others and to evaluate the clinical translation of expected health benefits of treatment with GlucoRegulate in further clinical studies. This is planned in future research.

### 4.4. Further Research and Clinical Evaluation of GlucoRegulate

GlucoRegulate is a promising scientifically based and clinically evaluated dietary supplement to support the treatment of insulin resistance and improving fasting and mealtime plasma glucose. Further experimental research is planned to investigate the mechanism of action of GlucoRegulate in improvement of metabolic, vascular health and renal health [5,86], including the recent hypothesis that GlucoRegulate may support improvement of the impaired incretin effect in obesity and diabetes for improved control of blood glucose and appetite [31]. We are now planning further phase 2 studies to evaluate sustained correction of insulin resistance and improvement of glycemic control in subjects living with obesity and/or prediabetes and studies to evaluate the effectiveness of GlucoRegulate in the prevention and remission of early-stage T2DM. A further target for clinical evaluation is treatment of early-stage MASLD, which has high global prevalence of 38% and associated increased risk of cardiovascular disease and progression to metabolic dysfunction-associated steatohepatitis (MASH), fibrosis, cirrhosis and MASH-related hepatocellular carcinoma—reviewed in [21,93,94]. There is currently no treatment for MASLD [95]. Interestingly, improvement of insulin resistance assessed by the oral glucose insulin sensitivity index (OGIS) is considered to be an indicator of improvement of MASLD [96]. OGIS was used in the HATFF study, which GlucoRegulate increased [1]. Additional applications for GlucoRegulate are treatment of early-stage osteoarthritis [97], prevention of age-related decline in respiratory function where insulin is considered to be a risk factor [98] and increased insulin sensitivity for improved athletic performance, *cf*. increase in insulin sensitivity by physical exercise assessed by OGIS [99,100].

## 5. Conclusions

The precision dietary supplement, GlucoRegulate, developed to increase expression of Glo1 and counter dicarbonyl stress, is associated with a range of cytoprotective health benefits. These include protection against dicarbonyl stress and activation of the UPR, lipid peroxidation, oxidative stress, hyperglycemia-linked glycolytic overload, proteotoxicity and low-grade inflammation. It offers a safe and effective dietary supplement strategy to support the treatment of insulin resistance, prevention of T2DM, treatment of early-stage MASLD and healthy aging.

## Figures and Tables

**Table 1:** Chemical structure, main natural sources and biosafety evaluation of (a) *trans*-resveratrol, (b) hesperetin and (c) GlucoRegulate.

Property	Comment		References
(a) *trans*-Resveratrol			
Chemical name and molecular structure	3,5,4′-trihydroxy-*trans*-stilbene		
	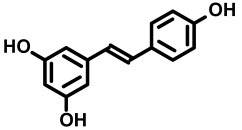		
Natural sources	Japanese knotweed (*Polygonum cuspidatum*), grape vines—stem, leaves and berry skin (*Vitis* spp.) and pistachio nut (*Pistacia vera*); typically 50, 2 and 0.2 mg/100 gm fresh weight, respectively. Lower contents in blueberry, bilberry, cranberry (*Vaccinum* spp.) and mulberry (*Morus* spp.)		[23,24]
Safety assessment	European Food Safety Authority (EFSA) Panel considered that the human studies indicate no adverse effects below 1 g tRES per day. tRES up to 750 mg/kg/day for 3 months in rabbits and rats was well tolerated, non-toxic with no effect on reproductive capacity in male or female rats and no embryo fetal toxicity. US Food and Drug Administration preliminary review supported the use of up to 3 g tRES per day.		[25,26,27]
	(b) Hesperetin		
Chemical name and molecular structure	(2*S*)-3′,5,7-Trihydroxy-4′-methoxyflavan-4-one		
	* 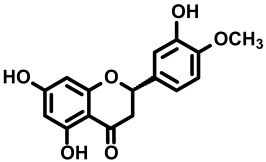 *		
Natural sources	Citrus fruits: grapefruit, lemons, oranges, tangerines (0.3–27 mg/100 g fresh weight)		[27]
Safety assessment	Hesperetin has no mutagenic activity and a good safety profile. Toxicology assessment in mice gave LD_50_ > 5000 mg/kg by oral administration and is classified as safe.		[28]
(c) GlucoRegulate (tRES+HESP combination)			
Chemical name and molecular structure	*trans*-resveratrol (tRES)	Hesperetin (HESP)	
	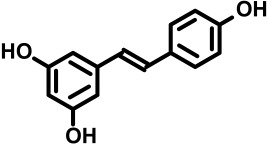	* 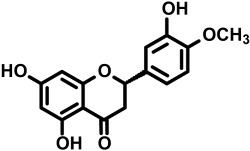 *	
Safety assessments	Dosing: 90 mg tRES and 120 mg HESP oral capsule, once daily, for 8 weeks. Clinical assessments: physical electrocardiogram and blood markers—aspartate aminotransferase, alanine aminotransferase, γ-glutamyl transferase, alkaline phosphatase, albumin, total protein, bilirubin, hemoglobin, red blood cell count, mean corpuscular volume, leukocyte count, thrombocyte no, prothrombin clotting time. All were normal.		[1]

**Table 2:** Proposed target pharmacology and health-beneficial responses of GlucoRegulate.

Target Pharmacology	Health Beneficial Response	References
Increased expression of Glo1	Decrease in methylglyoxal (correction of dicarbonyl stress) Prevention of activation of UPR, endoplasmic reticulum stress and low-grade inflammation Prevention of cell detachment from extracellular matrix and decreased angiogenesis for improved improving wound healing Therapeutic application: insulin resistance, prediabetes, diabetes, renal disease, cardiovascular disease and healthy aging	[1,3,8,11,32,33]
	Decreased methylglyoxal-mediated mutagenesis Therapeutic application: cancer prevention	[10,34]
Increased expression of G6PD	Decrease in cellular glucose-6-phosphate concentration Prevention of activation of ChREBP/Mondo A for decreased expression of glycolytic and lipogenic gene expression, countering glycolytic overload, impaired incretin effect and steatosis Increased metabolic flux into pentosephosphate pathway for NADPH to support biosynthesis and antioxidant mechanisms Therapeutic application: insulin resistance, prediabetes, diabetes, renal disease and MASLD	[1,3,8,31,32]
Decreased expression of SREBP1 and FASN	Decreased lipogenesis—decreased hepatic steatosis and ectopic fat deposition Therapeutic application: MASLD	Under investigation

Abbreviations: ChREBP/Mondo A, carbohydrate response element binding protein and its mainly extra-hepatic paralogue; G6PD, glucose-6-phosphate dehydrogenase; Glo1, glyoxalase 1; MASLD, metabolic dysfunction-associated steatotic liver disease; UPR, unfolded protein response.

**Table 3:** Receptors for *trans*-resveratrol and hesperetin in cell responses. Key: green shading—effects at clinically achievable concentrations; pink shading—effects likely not clinically translatable.

Receptor	Effective Concentration	Signalling Effect	Reference
***trans*-Resveratrol**			
Nrf2 activator	EC_50_ = 2.5 µM	Glo1 inducer, antioxidant, anti-inflammatory, anti-glycolytic overload and enhanced proteostasis in vitro	[1]
Phosphodiesterase-1 inhibitor	IC_50_ ≈ 6 µM	Increased cAMP in C2C12 myotubes	[76]
Aryl hydrocarbon receptor antagonist	IC_50_ ≈ 5 µM	May increase energy expenditure (brown fat UCP1-linked thermogenesis and muscle fatty acid β-oxidation)	[56,77]
F0F1-ATPase/ATP synthase inhibitor	IC_50_ ≈ 18.5 µM	Increased cellular ADP/ATP ratio. Activation of AMPK	[78,79]
Phosphoinositide 3-kinase inhibitor	IC_50_ ≈ 25 µM	Competitive inhibition at ATP binding site. Insulin resistance	[80]
Exchange protein directly activated by cAMP-1 activator	Agonism 50 µM	Activation of AMPK	[76]
Mitochondrial permeability transition pore-linked apoptosis	50–200 µM	Release of cytochrome c from mitochondria. Apoptosis. Cytotoxicity to tumor cell lines in vitro.	[81]
**Hesperetin**			
Nrf2 activator	EC_50_ = 0.6 µM	Glo1 inducer, antioxidant, anti-inflammatory, anti-glycolytic overload and enhanced proteostasis in vitro	[1]
Protein kinase A activator	Agonism 1 µM	Activation of sirtuin-1 and fyn kinase	[4,82]
Aryl hydrocarbon receptor antagonist	Antagonism ≥ 1 µM	May increase energy expenditure (brown fat UCP1-linked thermogenesis and muscle fatty acid β-oxidation)	[57]
Phosphodiesterase-4	K_i_ = 46 µM	Anti-inflammatory effects	[83]
Bax-linked mitochondrial pathway of apoptosis	IC_50_ = 70 µM	Cytotoxicity to tumor cell lines in vitro.	[84]
***trans*-Resveratrol and hesperetin**			
Nrf2 activator	EC_50_ = 1.5 µM tRES (+5 µM HESP)	Glo1 inducer, antioxidant, anti-inflammatory, insulin sensitizer, prevents glycolytic overload and proteotoxicity in vivo	[1,11]

## Data Availability

Data from this study are available from the corresponding author upon reasonable request.
